# Supplementary material for: Key components of post-diagnostic support for people with dementia and their carers: A qualitative study
Source: PLoS One. 2021 Dec 20;16(12):e0260506. doi: 10.1371/journal.pone.0260506 (PMC8687564; doi:10.1371/journal.pone.0260506)
Supplement: S2 File — (DOCX) [file pone.0260506.s002.docx]

# **PriDem topic guides**

**PriDem Phase 1 topic guide (commissioners and service managers)**

Introduction, review of consent, confirmation of permission to audio record the interview

**SECTION 1 – DETAILS OF THE SERVICE**

1.0 Can I start by confirming your job title and the name of the service you work for?

1.1 Can you tell me about the service you provide/commission for pwd and their families?

1.2 Thinking about the whole of the dementia trajectory, what do you see as the main gaps in service provision for a) pwd and b) families/carers in your area?

**SECTION 2 – RECOMMENDATIONS IN RECENT NICE GUIDELINES**

The recent NICE guidance recommended:

1. people with dementia should have a single named health or social care professional who is responsible for coordinating their care
2. cognitive stimulation therapy
3. carer psychoeducation.

- Explore the extent to which these areas are already covered by the service
- Perceived benefits and who should provide it
- Where do you think this should be based and why
- Skills and knowledge needed for the role
- Facilitators and barriers to implementation

**SECTION 3 – SHIFTING THE FOCUS TO PRIMARY CARE LED OR CO-ORDINATED SERVICES**

For many chronic conditions, most of the care most of the time is now provided by primary care, for example through specialist nurses and regular follow up.

3.1 Do you think this is appropriate for dementia? And why?

3.2 What do you think are the potential benefits of this approach?

- 1. What are the facilitators and barriers to this approach?
  2. What might this look like in practice?
  3. Are there components that you think could not be done by primary care?

3.6 What would primary care need to be able to deliver this?

**SECTION 4 – WRAPPING UP**

Is there anything else you would like to say about either your service or other services for people with dementia?

**PriDem Phase 2 topic guide (frontline staff)**

Introductions, review of consent, confirmation of permission to audio record the interview

**SECTION 1 – EXPERIENCES OF DELIVERING POST DIAGNOSTIC SUPPORT**

- 1. What do you think are the most positive / unique aspects of the service?

1.2 If we were going to roll out your service more widely, can you share your views on

- Key components of the model
- Staff skills, training and supervision needed to make it work
- Why this approach works in your local context, and how it might need to be adapted
- Gaps / areas for service development

**SECTION 2 – LINKS WITH PRIMARY CARE**

2.1 How does your service work with primary care? *Prompt for:*

- Communication e.g. shared patient record?
- Day to day contact
- How could links be improved? And if they did improve, how would this impact on a) service users and their outcomes, b) professionals themselves?
- If linked to MDT meetings – who attends, when, how are they involved?

**SECTION 3 – RECOMMENDATIONS IN RECENT NICE GUIDELINES**

The recent NICE guidance recommended:

1. people with dementia should have a single named health or social care professional who is responsible for coordinating their care
2. cognitive stimulation therapy
3. carer psychoeducation.

- Explore the extent to which these areas are already covered by the service
- Perceived benefits and disadvantages
- Facilitators and barriers to implementation
- If this service was commissioned locally, how might it impact on your service?

If already provided at service,

- Explore key skills/knowledge needed for the role
- Explore views on who should provide (NICE suggests mid-point band 6 nurse)

**SECTION 4 – WRAPPING UP**

Is there anything else you would like to say about either your service or other services for people with dementia?

**PriDem Phase 2 topic (service users)**

Introduction, review of consent, confirmation of permission to audio record the interview

**SECTION 1 – CURRENT SERVICE USE**

1.1 Can you give me a picture of the help you / you and your relative have had, from diagnosis of memory and thinking problems to the present day? *For each element, prompt for:*

- What do you like about the service? Anything you dislike / think could be improved?
- Was the timing of the service / help you received right?
- What difference services make to your life (and to your relative’s life)
- Whether and how services are tailored to your needs
- How well do different services work together – including with your GP

1.2 What have been the main gaps in the services or help you have received?

**SECTION 2 – VIEWS ON POST DIAGNOSTIC SUPPORT**

2.1 To explore preferences for organisation and access to post diagnostic support including:

- Areas to be covered by post-diagnostic support
  This could include:
  - Being as independent as possible
  - Making sure you are safe
  - Looking after your physical health
  - Keeping up with hobbies/interests
  - Understanding your problems and how best to manage them
  - Other areas?
- How should contact be made (e.g. proactive, reactive, frequency)?
- Place of appointments (e.g. own home, community centre, GP surgery)?
- Having a named individual as a point of contact or a team/hub?
- How to access advice?

2.2 To explore views and experiences of annual dementia review by GP including:

- Whether they have had a review with a GP about dementia?
- What do you like about the review? Anything you dislike / think could be improved?
- Was the timing of the review right?
- Whether and how the review was tailored to needs?
- How often do you think these reviews should happen? When should the first review be?

**SECTION 3 – WRAPPING UP**

Is there anything else you would like to tell us about the support you have received for your problems with memory and thinking?
