# Supplementary material for: Key components of post-diagnostic support for people with dementia and their carers: A qualitative study
Source: PLoS One. 2021 Dec 20;16(12):e0260506. doi: 10.1371/journal.pone.0260506 (PMC8687564; doi:10.1371/journal.pone.0260506)
Supplement: Attachment — Submitted filename: Response to reviewers.docx [file pone.0260506.s003.docx]

## **Response to reviewer comments**

|  | Journal requirements | Response |
| --- | --- | --- |
| 1. | Please ensure that your manuscript meets PLOS ONE's style requirements, including those for file naming. The PLOS ONE style templates can be found at  <https://journals.plos.org/plosone/s/file?id=wjVg/PLOSOne_formatting_sample_main_body.pdf> and  <https://journals.plos.org/plosone/s/file?id=ba62/PLOSOne_formatting_sample_title_authors_affiliations.pdf> |  |
| 2 | Please include additional information regarding interview guide used in the study and ensure that you have provided sufficient details that others could replicate the analyses, and please include a copy as Supporting Information. | Topic guides have been included as supporting information and a summary provided in the paper. |
| 3 | We noted in your submission details that a portion of your manuscript may have been presented or published elsewhere.  [We have published two papers drawing on the same datasets. There is some overlap with the methods sections. The focus of the first paper was on professionals' views of a task-shifted or task-shared approach. The second paper explored barriers and strategies to the delivery of post-diagnostic support. No quotations in the present paper have previously been published and this paper contains a greater focus on the views of people with dementia and carers than the earlier papers. There is some overlap between the barriers to post diagnostic support and the components; however, the barriers paper focuses more on how services are delivered, whereas this paper explores what components are being delivered. We believe this paper is distinctly different to the previous publications and adds to the existing literature.]  Please clarify whether this publication was peer-reviewed and formally published. If this work was previously peer-reviewed and published, in the cover letter please provide the reason that this work does not constitute dual publication and should be included in the current manuscript. | We would like to clarify that it is not the case that a portion of this manuscript has been published elsewhere. Rather this paper is one of a series of outputs reporting different aspects of an extensive qualitative dataset, which could not have been reported coherently as a single paper.  The methods section is similar to previously published peer reviewed papers in the series, but has been rephrased so that it is not identical. The published papers have been submitted with this paper. |
| 4 | We note that you have stated that you will provide repository information for your data at acceptance. Should your manuscript be accepted for publication, we will hold it until you provide the relevant accession numbers or DOIs necessary to access your data. If you wish to make changes to your Data Availability statement, please describe these changes in your cover letter and we will update your Data Availability statement to reflect the information you provide | We are currently making arrangements to make the data available but need to make some additional changes to the data files to ensure that the confidentiality of sites in Phase 2 is maintained. Once this has been completed, we will provide the relevant DOI. |
| 5 | One of the noted authors is a group or consortium [PriDem study team]. In addition to naming the author group, please list the individual authors and affiliations within this group in the acknowledgments section of your manuscript. Please also indicate clearly a lead author for this group along with a contact email address. | These details have been added to the acknowledgements section. |
|  | Additional Editor Comments: |  |
| 6. | The reviews are in general favorable and suggest that, subject to major revisions, your paper could be suitable for publication. Please consider these suggestions, and I look forward to receiving your revision. Please organize your qualitative study in accordance with the consolidated criteria for reporting qualitative research (COREQ) and the standards for reporting qualitative research (SRQR), and then upload completed forms as supplementary files.  [Note: HTML markup is below. Please do not edit.] | We had followed the SRQR in the paper and have now added the COREQ as a supplementary file, adding details to the text as needed. |

5. Review Comments to the Author

|  | Reviewer #1 | Response |
| --- | --- | --- |
| 8. | This is a very interesting paper and in-depth qualitative approach trying to understand the different components of post-diagnostic dementia care in England and Wales. Overall, this paper offers important new insights.  However, there are some minor and major issues which need addressing, including lack of participant data, inappropriate discussion of findings in the results section, lack of mentioning of social care in the Introduction and more broadly at the end of the paper, lack of discussion of key findings in the Discussion. Further details are below: | Thank you for these positive comments and your suggestions for improving the paper. |
| 9. | Whilst the authors provide a very clear and in-depth background about the structure of care in the UK, they miss out social care and discussing the interface between health and social care - after all, social care is the key form of care that people with dementia should access after a diagnosis (if they are aware of services, get referred, have enough funds, find the services suitable, etc...). The authors should therefore clearly refer to social care in the introduction, as dementia care is not solely run by primary care. | Thank you for highlighting this unintentional bias. We have revised the introduction and included more emphasis on the range of sectors involved in post diagnostic support and the interface between them. |
| 10. | In the last paragraph of the intro, the authors should state what type of study they are conducting, as opposed to stating in the last sentence 'The aim of this paper...'. | The last paragraph of the introduction has been revised to include a description of the type of study (which we have now elaborated) and the aim. We hope this resolves the issue. |
| 11. | How were topic guides developed? Were people affected by dementia involved? What was the broader level of public involvement in the study? This is not mentioned and a lack of public involvement would be a clear limitation. | Further details of public involvement have been added. In brief, the topic guides were reviewed by our mixed stakeholder panel. This panel also reviewed the components. |
| 12. | Where are the background characteristics of participants? They are not stated or referred to in the results. | We have added tables summarising characteristics of participants. |
| 13. | There are no page numbers by the way. | Page numbers have now been added. |
| 14. | Under timely identification and management of current & future needs, the authors discuss the finding, which is not suitable for the results and sentences including references should be removed [lines 199-201]. | These lines have been deleted. |
| 15. | Under the following theme, it seems strange that there was no mention of other non-cognitive symptoms. Instead, there is only a focus on cognition and behavioural symptoms, as well as functional deficits at one point, but no mention of for example speech difficulties, or motor or vision problems, as occur in certain subtypes. | Thank you for highlighting this omission. We have now referred to additional non-cognitive problems as you suggest. Sensory impairment has now been included in physical health. |
| 16. | Again, picking up on a previous comment, the authors should not refer to existing literature in the field, discuss their findings, or provide further background in the results section. please delete lines 301-303. | These lines have been deleted. |
| 17. | Is the named point of contact and the care coordinator not effectively the same, or at least should be? Otherwise again there would be too many names/people to remember to contact, whereas accessing and utilising post-diagnostic dementia care should be as simple and straightforward as possible. | We have purposefully distinguished between these two roles. While we appreciate that the UK NICE guidelines combine the two roles, this model was rarely seen in practice. Further, there was a lack of shared understanding of care coordination among professionals (Wheatley et al 2020). While most professionals interviewed agreed that a named point of contact was valuable for all people with dementia and carers, many argued that care coordination was only required for relatively small numbers of people with dementia, often for time-limited periods when needs were complex and multiple agencies were involved. Providing a care coordinator for all people with dementia and carers, many of whom receive few services, was seen by many professionals as an inappropriate use of scarce resources.  We have therefore followed the model adopted successfully in a number of sites and distinguished between these two roles. A named point of contact, often an unregistered member of staff, was typically involved in assessment of needs, signposting, support of non-complex needs, and ongoing review. The role was supplemented in some services, with a step-up/down model whereby people with complex needs received time-limited intensive support from a care-coordinator (typically a registered nurse or allied health professional). We have reviewed the text and are not sure how we could clarify this further. |
| 18. | The authors do not discuss the findings in depth in the Discussion, but provide a broad brush stroke of stating that this is a new guidance for a holistic PDS model. | We have extended the discussion whilst being mindful of the length of the paper. We have now linked the findings to existing work on outcomes as well as strengthening links to literature relating to our two overarching themes. |
| 19. | Furthermore, there is no mentioning of the pandemic. Yes, data were collected prior to the pandemic, yet COVID-19 has flipped PDS on its head and made it even more difficult to access care. Thus, the authors should refer to evidence in the field of how the pandemic has affected social care in dementia and possible implications for the long-term. | Thank you for this observation. We have added a paragraph to the discussion about COVID-19. |
|  | Reviewer #2 |  |
| 20. | This is a well written, interesting article on a very important topic. There are some comments I have made for the authors to consider – but they are minor and I recommend the editor publish this article once the comments have been addressed.  On a personal note, it’s nice to review such a well put together paper after reviewing so many dire ones of late – so thank you! | Thank you |
| 21. | There needs to be researcher reflexivity in this article. I realise the interviews and analysis were conducted a while ago, but this is, in my opinion, not something that can be glossed over in qualitative research. So, how do the researchers feel their own practices, judgements, belief systems affect the data collection and analysis? How do you position yourselves in the research regarding your own background, work experience and other appropriate experiences? All this would add trustworthiness to the article and give it more depth. You could add this to the discussion – not much necessarily, but it needs adding and making clear. | We have added a new section on reflexivity to the methods with additional information on the backgrounds of the researchers involved in data collection and analysis. We have also commented on the trustworthiness of the data in the discussion. |
| 22. | A literature review was very recently published on integrated dementia care titled ‘Integrated dementia care: A qualitative evidence synthesis of the experiences of people living with dementia, informal carers and healthcare professionals’. Seeing as it is so relevant to this article and its recent publication, it would make sense to include here how your study extends knowledge from that review and how they link together. However you decide to address this comment, I feel the above article does need citing in your paper. | Thank you for drawing our attention to this recent review. We have read the paper carefully and cited it in the introduction and discussion. |
| 23. | I am really very glad that you interviewed people living with dementia and included some of their comments in your findings section. There is so much research out there on similar topics where the voice of people living with dementia is missing completely, so I appreciated this. I often get very irritated when authors make recommendations for improving/changing systems or approaches to dementia care when only informal carers (also important) and health professionals have been consulted……or worse still just health professionals or academics. You interviewed a huge number of participants from diverse backgrounds and professions, informal carers and people living with dementia – I would add this to the ‘strengths and limitations’ section in the discussion of your article, but feel free to ignore this point if you feel it’s a bit OTT. | Thank you for this comment. It is useful to get this feedback as we were disappointed not to involve more people living with dementia and this is probably why we did not think of including it as a strength of the study. |
| 24. | I am not very good at spotting typos (sorry!) – so hopefully the other peer reviewer(s) will have picked any up. But as I said previously, this is very well written paper so I doubt there are many. | Thank you. We have run a check of spelling and grammar on the manuscript again to try to ensure that any errors have been corrected. |

[NOTE: If reviewer comments were submitted as an attachment file, they will be attached to this email and accessible via the submission site. Please log into your account, locate the manuscript record, and check for the action link "View Attachments". If this link does not appear, there are no attachment files.]

While revising your submission, please upload your figure files to the Preflight Analysis and Conversion Engine (PACE) digital diagnostic tool, [https://pacev2.apexcovantage.com/](https://eur03.safelinks.protection.outlook.com/?url=https%3A%2F%2Fpacev2.apexcovantage.com%2F&data=04%7C01%7Cclaire.bamford%40newcastle.ac.uk%7Cf15994b5ab47405a0f0608d96f03982c%7C9c5012c9b61644c2a91766814fbe3e87%7C1%7C0%7C637662884706387414%7CUnknown%7CTWFpbGZsb3d8eyJWIjoiMC4wLjAwMDAiLCJQIjoiV2luMzIiLCJBTiI6Ik1haWwiLCJXVCI6Mn0%3D%7C1000&sdata=bqWtmqKa6T7SAhbn5rfPyPLPo9QglV9CdK044ldOc84%3D&reserved=0). PACE helps ensure that figures meet PLOS requirements. To use PACE, you must first register as a user. Registration is free. Then, login and navigate to the UPLOAD tab, where you will find detailed instructions on how to use the tool. If you encounter any issues or have any questions when using PACE, please email PLOS at [figures@plos.org](mailto:figures@plos.org). Please note that Supporting Information files do not need this step.
